# The Use of Purified and Modified Cellulose in Wine Production and Analysis

**DOI:** 10.1111/1750-3841.71336

**Published:** 2026-08-06

**Authors:** Stephan Sommer

**Affiliations:** ^1^ Department of Food Science University of Tennessee Knoxville Tennessee USA

## Abstract

Cellulose is not only one of the most abundant biological macromolecules but can also be purified and modified to serve a multitude of purposes in industrial winemaking processes. Being composed of only glucose and naturally occurring unbranched in nature as strands and fibrils, cellulose can be used for filtration directly or as a filtration aid. It can also be enriched to selectively remove unwanted compounds from wine or chemically modified to change its solubility, hydrophobicity, and reactivity properties. The most common examples in wine production are methylcellulose, carboxymethyl cellulose, and dicarboxymethyl cellulose. With the exception of methylcellulose, these compounds are mostly used for achieving various forms of physical stability, such as cold and heat stability. Methylcellulose, while not allowed as an additive in wine, can be used for analytical purposes to quantify condensed tannins in a variety of matrices. This review covers the wine‐related uses of purified cellulose by itself and its variations in wine filtration, as well as the chemical modifications and uses of cellulose gum in the winemaking process and for wine analyses. The sustainability of cellulose compared to other fining agents, filtration aids, and additives makes it a versatile and viable option in the world of wine.

## 1 Introduction

Cellulose in its native form is a naturally occurring biopolymer composed of β‐1,6‐linked glucose molecules with an average degree of polymerization between 100 and 20,000 (Y.‐H. P. Zhang and Lynd 2005), resulting in a source‐dependent molecular weight between 30 and 3000 kDa (Battista 1944; Berggren et al. 2003; Burchard 2003). Due to this unique bond, cellulose forms long strands that combine into fibrils and eventually into fibers. That makes cellulose one of the most important structural materials in plants (Somerville 2006) and, therefore, an abundant renewable resource. While pure cellulose does not naturally occur by itself and is usually accompanied by hemicellulose and lignin (Hallac and Ragauskas 2011), it is the perfect biopolymer for technical applications due to its chemical and physical properties. Especially the straight, unbranched nature of the cellulose molecule allows for technical and chemical manipulation without substantial steric hindrance. Once purified, cellulose is a homogenous material only composed of glucose molecules, which means that its solubility, hydrophobicity, and three‐dimensional structure can be precisely adjusted to many specific applications (Burchard 2003).

Due to the overall molecular size and hydrophobicity of cellulose, it can be used to immobilize microorganisms (Agouridis et al. 2008), deliver biomedical treatment by carrying medications (Sun et al. 2019), or carry any other soluble compounds that function as a production aid in an industrial process but are not classified as additives (García‐Ríos et al. 2018). In order to increase solubility and add functionality, cellulose can be chemically modified. Within the right pH range and concentration, modified cellulose forms a gel and increases the viscosity of an aqueous solution (Keller 2020). This increases the number of possible applications in various industries, including food, medical, and industrial solutions (Du et al. 2011; Hebeish et al. 2013; Keller 2020; Salama 2018). These modifications are mostly chemical in nature and involve the addition of side chains to the homogenous strand. The resulting products, for example, methylcellulose (MC), carboxymethyl cellulose (CMC), or dicarboxymethyl cellulose (DCMC), display different physical and chemical properties in terms of solubility, hydrophobicity, and reactivity, depending on their molecular sizes and degrees of substitution (Burchard 2003; Keller 2020; Schwendig and Thier 1989).

The most common methods to modify the physical and chemical behavior of cellulose are based on dissolution of the tightly packed fibers by swelling the material and substitution of side chains in the molecule. While purified cellulose is not water soluble, the fibrils can be separated by steeping them in sodium hydroxide (Keller 2020). The introduction of functional groups through etherification decreases hydrophobicity and alters the reactivity. Depending on the intended use, the selection of the functional group, as well as the degree of substitution, determines the properties of the modified cellulose. This makes modified cellulose one of the most promising additives and fining agents in wine production due to its flexibility and sustainability. The properties can be adjusted as required by the planned application, and the raw material cellulose is a regenerative substrate that can be isolated from plants or even waste material (Harini et al. 2024; Paradelo et al. 2013). Instead of being mined like bentonite or perlite commonly used in wine production, it can be regrown and is biodegradable after use, eliminating the need for expensive and unsustainable disposal.

The objective of this review is to present the traditional as well as innovative uses of cellulose and its modifications in the production and analysis of wine.

## 2 Cellulose

### 2.1 Use of Cellulose in Wine Production

**FIGURE 1:**
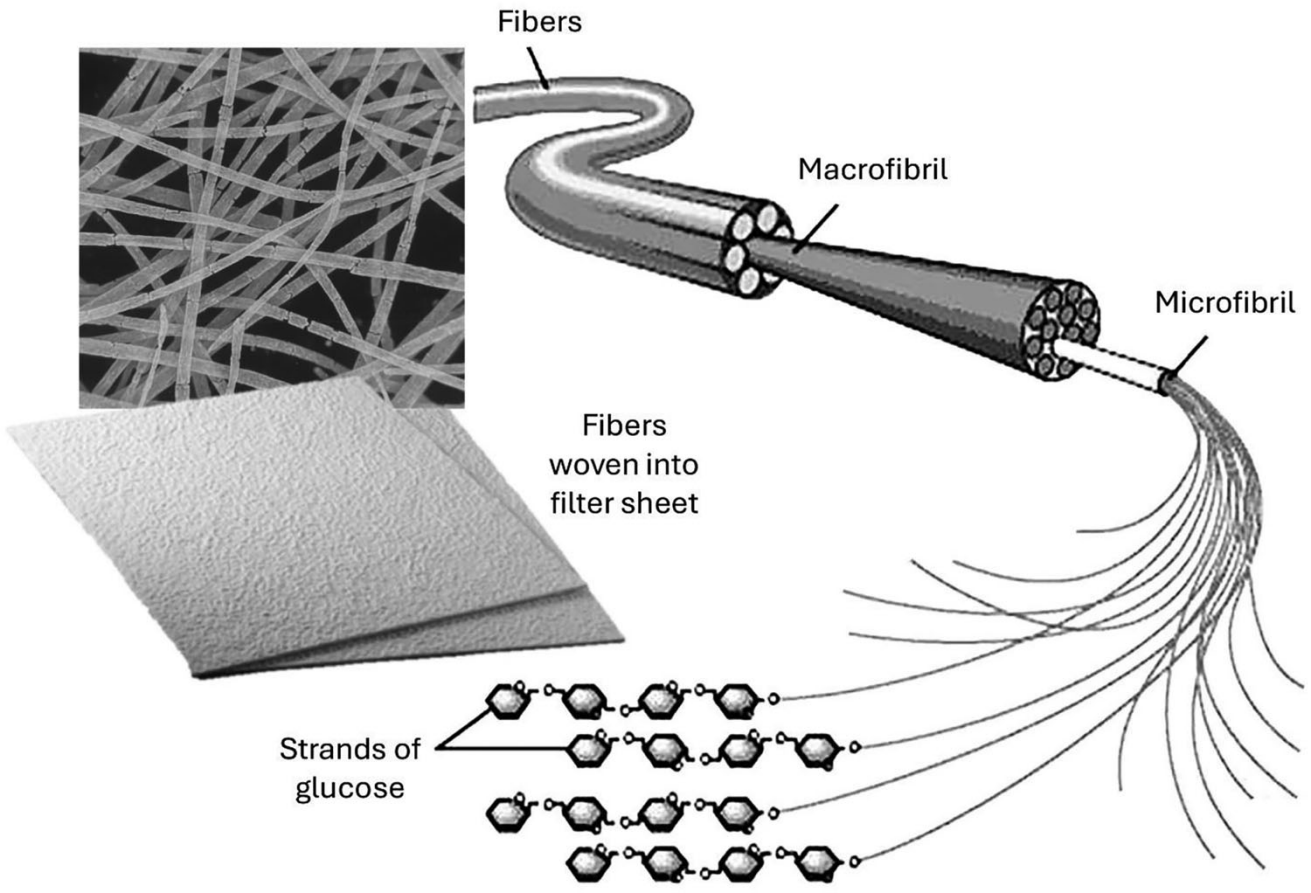
Structure of cellulose strands, fibrils, and fibers. Fibers can be woven into sheets for filtration (adapted from Ferreira Alves do Prado et al. [2011]).

Purified cellulose has been used in wine production for a very long time (Master 2024a; Saywell 1935). Due to its absorbent qualities and fibrous structure, filter sheets and filter aids are the most common applications. Figure 1 describes the general structure of cellulose from the smallest unit, strands of glucose molecules, to fibrils and fibers and finally into fiber bundles (Ferreira Alves do Prado et al. 2011).

Those fiber bundles can then be woven and compressed into sheets or any other densely compacted network. While cellulose filters show more pore size variability compared to synthetic materials, the sheets can still achieve nominal filtration close to sterile conditions for wine, as suggested by the manufacturers. Table 1 summarizes the filtration categories and pore sizes of the main commercial cellulosic wine filter sheets. Manufacturers use different ratings to classify their cellulosic filter sheets as coarse, fine, and polishing filtration based on the average pore size and resulting particle size retention. While coarse filtration removes particles that are visible to the naked eye, fine and polish filtration remove objects in the lower micrometer range. Below 0.45 µm average pore size, the filtration step removes microorganisms such as yeast and bacteria, making the wine more shelf stable.

**TABLE 1:** Average pore sizes of the most common filter sheets used for wine filtration, sorted by manufacturer, including their product codes. All specifications are taken from manufacturers’ information (FILTROX Carlson Ltd., St. Gallen, Switzerland; Eaton Filtration LLC., Tinton Falls, NJ, USA; Gusmer Enterprises Inc., Mountainside, NJ, USA; Seitz/Pall Corporation, Port Washington, NY, USA).

Application	Manufacturer				Average retention rating [µm]
	Carlson	Eaton	Gusmer	Seitz/Pall	
Coarse filtration	XE10H	BECOPAD580	1315SD	K900	9–10
	XE20H		1325SD	K800	7–8
Polishing filtration	XE70H		1330SD	K700	5–7
	XE90H		1340SD	K300	3–4
	XE150H	BECOPAD550	1345SD	K250/ZD10	2.5
Haze filtration	XE200H		1350SD	K200	2.0
	XE280H	BECOPAD450	1355SD	K100/ZD10	1.0
Polishing filtration	XE400H	BECOPAD350	1950SD	KS80/ZD08	0.8
	XE550H	BECOPAD270	1960SD	KS50	0.5
Nominal sterile filtration	XE675H	BECOPAD220	1965SD	EK/ZDEK	0.45
	XE900H	BECOPAD170	1970SD	EK I	0.35
	XE1200H	BECOPAD120	1975SD	EKS	0.25

Traditionally, cellulose is purified from lignocellulosic biomass or waste material using a combination of sodium hydroxide and bleach after delignification (Amarasekara et al. 2020; Sundarraj and Ranganathan 2018). The yield varies depending on the substrate as well as the extraction conditions, which can be chemically harsh and decrease the quality of the final product. A newer study modified the purification protocol and was able to retain a higher degree of polymerization in the purified cellulose, potentially broadening its use in industrial applications (Hivechi and Bahrami 2016). While cellulose can be purified from all plant material and a variety of waste materials, it is required to be highly purified to be used in food. Food‐grade cellulose is usually derived from wood pulp or cotton and has to meet strict standards in terms of purity and color (Lavanya et al. 2011). While the same standards apply for cellulose that is used in wine filtration, the fibers must be from deciduous and coniferous trees and long enough and fibrillated to be pressed into filter sheets. Some manufacturers add diatomaceous earth or perlite to the cellulose pulp for additional structural integrity and improved wet strength (Master 2024c). Additional strength can be achieved with approved resins such as polyamidoamine at concentrations up to 4% of the dry cellulose fiber weight (defined in food safety standards such as EU regulation № 2019/934).

Wine needs to be filtered for a variety of reasons, ranging from the removal of suspended particles to achieving stability and extending shelf life. This includes the removal of macromolecules such as protein, polyphenols, and polysaccharides (Mierczynska‐Vasilev and Smith 2015). Most of these objectives can be achieved by cellulose filtration alone; however, other filter aids such as perlite and diatomaceous earth, in combination with closed canister filters, are alternative techniques that are widely used (Catania et al. 2010). While the aforementioned methods do not provide absolute microbial stability due to a higher variability in pore size, cellulosic membrane filters can be used to remove microorganisms below 0.45 µm in size (Duarte et al. 2017). The manufacturing process of these membranes allows for a tighter network and a smaller pore size for filtration (Urkiaga et al. 2002).

Besides the compressed filter sheets, cellulose can also be used in powdered form as a filter aid (Berg et al. 1986; Braun et al. 2011). The surface area is vastly increased, allowing for the adsorption of particles and macromolecules; however, this technique requires a specialized filtration setup. While the cellulose is suspended in the liquid, similar to bentonite (Sommer and Tondini 2021), it has to be removed using a filter with increased capacity for solids, such as a lees plate and frame filter system. Powdered cellulose can then be used to improve the efficiency of lees recovery (Breniaux et al. 2021) or the clarification of microbially impacted and hard‐to‐clarify special wine styles such as late harvest and ice wines (Ma et al. 2020; Ridge et al. 2021). Another filtration‐related application of purified cellulose in wine is the use of tubular filters for cold pasteurization, a term used to describe the removal of microbial cells through filtration (Gialleli et al. 2015; Papafotopoulou‐Patrinou et al. 2016). Cellulose is typically purified from waste material such as sawdust and pressed into a filter with a small enough average pore size to effectively remove yeast and selected bacteria from wine. Similar to cellulosic filter sheets, the tubular cellulose (TC) cannot provide an absolute filtration due to inconsistencies in pore sizes; however, previous studies have found efficiencies over 90%, up to 100% (Gialleli et al. 2015). An additional advantage of TC is that it can be recovered multiple times by hot water rinses, increasing its value and sustainability as a filter aid and reducing the use of chemical cleaners.

It is worth mentioning that cellulose can be used as a carrier material for encapsulated microorganisms in food systems as well (Nedovic et al. 2011). A recent study on sparkling wine has shown that cellulose is an alternative to traditional immobilization materials due to its inert nature (Berbegal et al. 2019). Others used a cellulose sponge material to immobilize lactic acid bacteria for malolactic fermentation in wine (Maicas et al. 2001). The efficiency of immobilized cultures is comparable to traditional inoculation methods while providing technological advantages in terms of clarification and process control.

### 2.2 Modifications of Cellulose Filter Sheets

While purified cellulose has properties that make it usable for filtration by itself, it can also be enriched or fortified to selectively remove specific compounds from wine. Earlier applications decades ago, such as the addition of asbestos fibers to cellulosic filter sheets (Saywell 1935), were abandoned due to heavy metal leaching and toxicology concerns. More recently, studies have been done on heavy metal removal (Neira et al. 2022), mitigation of cork taint (Jung et al. 2008), and other off‐flavors (Philipp et al. 2022). Besides the additives for structural integrity and wet strength of the filter sheet, other selective fining agents can be incorporated for the added benefit of the filtration. One of the earliest examples was a specialized filter sheet that used an aluminum silicate to selectively remove 2,4,6‐trichloroanisole while not affecting the positive aroma properties of the wine (Jung et al. 2008). A different study confirmed the reduction of cork taint and also found that enriched cellulosic filter sheets can reduce the perception of geosmin, which is responsible for earthy and moldy aroma, while not affecting volatile phenols (Philipp et al. 2022). However, that study also found that positive aroma in wine can be impacted, especially esters and more volatile aroma compounds. Overall, the use of enriched filtration media has the potential to combine fining and filtration steps into one treatment. The use, however, should be targeted and tested for the reduction of mouthfeel and flavor that could negatively impact the wine.

## 3 Methylcellulose

**FIGURE 2:**
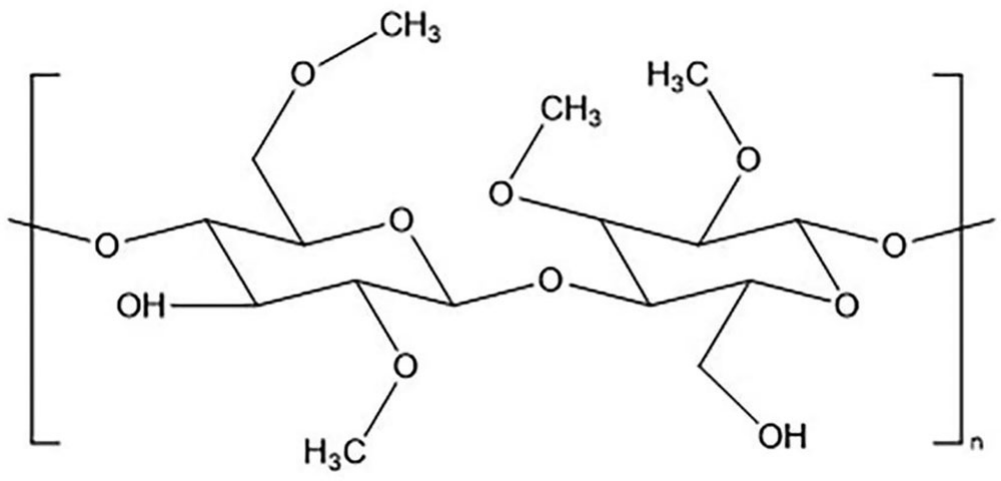
Molecular structure of a methylcellulose subunit.

While being quite common in baked goods (Grover 2022; Sanz et al. 2005), MC is not approved for use as an additive or technical aid in wine for human consumption. Therefore, it cannot be used at any point of the production process if the wine is to be sold commercially. However, its hydrophobicity and resulting reactivity with tannins make it a useful tool in wine analysis (Sarneckis et al. 2006). Figure 2 shows the structure of a molecular subunit of MC to illustrate the modification of the cellulose chain.

For more than 20 years, MC has been the preferred analytical tool for the quantification of condensed tannins in wine (Sarneckis et al. 2006). MC is used in the presence of ammonium sulfate to bind and precipitate tannins in wine, and the difference in absorbance at 280 nm before and after precipitation can be quantified as condensed tannins. The binding mechanism itself is fairly selective for polymeric polyphenols, since the methyl groups of the MC and the aromatic rings of the tannin molecule interact via hydrogen bonding. This type of interaction requires a larger number of reaction sites to form a strong bond and lead to an irreversible precipitation. A secondary mechanism involves hydrophobic interactions, which also require a large molecular size. Smaller polyphenols that might form a weak aggregate with MC will not permanently precipitate and will therefore not be included in the quantification. The ammonium sulfate eliminates proteins that could also absorb at 280 nm, making the spectrophotometric analysis more selective for tannins.

Several studies have compared the efficiency of tannin precipitation methods in wine (Harbertson and Downey 2009; Watrelot 2021; Wilhelmy et al. 2021) and apple cider (Bindon et al. 2023; Sommer et al. 2022) using MC and other selective reaction partners. The most common choice is bovine serum albumin (BSA) due to its homogenous molecular structure, inexpensive production, and high availability. However, as a protein, the functionality and performance strongly depend on the pH of the medium (Kongraksawech et al. 2007), making it a challenging choice in a highly acidic matrix such as wine. BSA was also shown to interact with other wine components (Graves and Sommer 2021) and requires special conditions to selectively precipitate polymeric polyphenols (Burken and Sommer 2025). This makes MC a more suitable choice for tannin precipitation from wine for analytical purposes.

## 4 Carboxymethyl cellulose

### 4.1 General Uses of CMC

**FIGURE 3:**
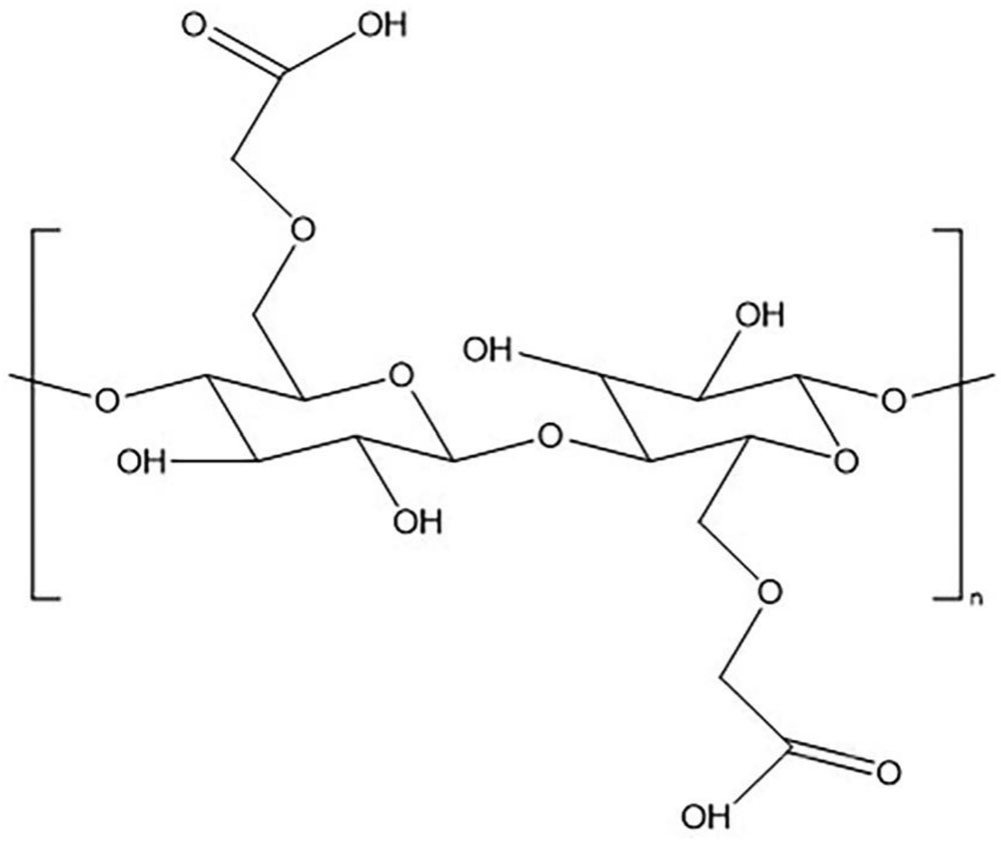
Molecular structure of a carboxymethyl cellulose subunit.

CMC was originally developed as an alternative to gelatin (Keller 2020) and has been used in food and consumer products for decades (Hollabaugh et al. 1945). The main functionality is based on its gel‐forming properties, making CMC a widely used thickening agent. The most common applications in terms of thickening activity are ice cream (Cheng et al. 2015), soup products (Chumlert et al. 2024; Y. Zhang et al. 2025), paper coatings (Du et al. 2011), toothpaste (Ahuja and Potanin 2018), other cosmetics (Costa et al. 2022; Kanlayavattanakul and Lourith 2010), and food products (Salari et al. 2017; Schuh et al. 2013). Other applications in biomedical engineering, waste treatment, and energy production are summarized in a recent technical review (Rahman et al. 2021). Figure 3 shows the general molecular structure of a CMC subunit.

The use of CMC in baked goods, similar to MC mentioned above, stands out in the long list of applications due to the versatility and specific interactions that shed light on the physical and chemical properties of CMC. It binds moisture effectively and “traps” it in the baked good, therefore influencing the texture, appearance, and shelf life of a wide variety of products (Keller 2020). Among the baked goods that have CMC on their ingredient list are cakes, icings, fillings, glazes, doughs, and, most importantly, chemically leavened cake mixes. CMC interacts with gluten proteins and prevents sugar and ice crystals from forming, which hints toward the main functionality when it is used in wine.

The technical properties of CMC dictate its uses and applications to a large degree. While the degree of polymerization and the resulting range of molecular weight determine the viscosity and hydrophobicity of a solution, the degree of substitution is important for solubility and stability. CMC with a high degree of substitution shows better solubility and stability properties (Batelaan et al. 1992), which makes it more suitable for medical and food applications. Table 2 summarizes the most important attributes for different applications to clarify the correlation between molecular structure and possible industrial uses.

**TABLE 2:** Specifications of commercial carboxymethyl cellulose preparations for different industrial applications, if characterized or specified.

Application	Molecular weight; MW [kDa]	Degree of polymerization; DP	Degree of substitution; DS	Reference
Food	Unspecified	Unspecified	0.60–1.25	S. Zhang et al. 2022
Wine	17–300	80–1500	0.60–0.95	Master 2024b
Cosmetics	<500	Unspecified	0.40–1.50	Costa et al. 2022
Technical	40–1000	100–3500	0.40–3.00	Keller 2020

### 4.2 CMC and Tartrate Stabilization in Wine

The earliest approved application of CMC in wine was for the stabilization of tartrate crystals in white and sparkling wine (Bliard et al. 2010; Lasanta and Gómez 2012). The method of choice to prevent potassium bitartrate crystals from forming in wine had been cold stabilization up to that point. For that treatment, wines are stored at or slightly below freezing (−4°C to 0°C) with or without seed crystals for an extended period of time (Coulter et al. 2015). This temperature range is chosen because it is slightly below the household refrigerator range, so crystals will not form when the consumer chills the finished and bottled product. Depending on the pH, ethanol content, potassium concentration, and organic acid balance, crystals will form at these low temperatures as the solubility reaches a minimum (Boulton et al. 1999). Seed crystals provide nucleation sites and speed up crystal growth. In some cases, the chilling capacity of the winery limits the ability of the winemaker to reach these low temperatures, or there are time restraints on the production process, which makes the use of additives for crystal stability a viable option. As described earlier, CMC has the ability to prevent crystal formation in baked goods (Keller 2020), so the functionality in wine is very similar. CMC forms a gel in aqueous solution, coating any crystals as a protective colloid and preventing them from growing (Coulter et al. 2015). It can also remove potassium ions from the reaction equilibrium, effectively stopping crystal growth (Ding et al. 2020). It is therefore a true ingredient in winemaking because it does not remove any of the crystal components but rather keeps them in solution while remaining in the wine itself (Guise et al. 2014).

Other additives that were introduced to the winemaking process, such as metatartaric acid (Cosme et al. 2024; Sprenger et al. 2015) and potassium polyaspartate (KPA) (Geveke and Runnebaum 2020; Martínez‐Pérez et al. 2020), also remain in the product but, in the case of metatartaric acid, have a limited shelf life. It is assumed to lose its protective effect (Sprenger et al. 2015) after approximately 9–12 months, limiting the use to wines intended for early consumption that sell out within that time. Another limitation is the export market since some countries do not accept imported wines that were treated with additives (Kubota et al. 2017). In terms of cost analysis, however, additives such as CMC, KPA, gum arabica, and metatartaric acid are very cost‐effective for stabilizing tartaric acid in wine (Lasanta and Gómez 2012). Only physical solutions such as ion exchange and electrodialysis are less expensive per volume, but the initial investment for this technology is cost‐prohibitive for small‐ and medium‐sized wineries. Interestingly, the traditional cold stabilization with or without seed crystals is the most expensive option due to high energy consumption (Low et al. 2008). This makes additives the best option for smaller wineries economically and from a technological perspective.

Some studies explored the shelf life of CMC and its functionality in wine over time (Gerbaud et al. 2010; Greeff et al. 2012; Marsh and Mills 2013). The results indicate that CMC has a longer lifespan in wine than metatartaric acid. The only loss in efficiency seems to occur when CMC is reacting with other wine components to form a precipitate (Claus et al. 2014; Sommer et al. 2016). In these cases, some of the CMC is lost by the precipitation reaction, lowering its efficiency as a protective colloid.

### 4.3 CMC and Proteins

CMC has been used in various studies over the years to isolate and purify proteins from different matrices (Hill 1978; Lali et al. 2000; Orwick‐Rydmark et al. 2016; Yu et al. 2004; Zadow and Hill 1978). CMC is known to interact with proteins at a high affinity rate depending on the pH and ionic strength of the medium, as well as the CMC‐to‐protein ratio (Hidalgo and Hansen 1971). While this selective affinity is helpful for biotechnology applications, it can lead to unexpected problems in a more complex matrix such as wine when CMC is used for an entirely different reason, such as tartrate stability.

In wine, the interaction of CMC with macromolecules was discovered as part of a problem that was previously reported by winemakers. The use of CMC in wine was limited to white and sparkling wines initially by the International Organization of Vine and Wine (Organisation Internationale de la Vigne et du Vin; OIV) (Gerbaud et al. 2010; Guise et al. 2014; Master 2010), since the addition causes a visible haze in some rosé and red wines (Sommer et al. 2016). Studies to investigate the mechanisms behind the turbidity found that the haze appeared in about 20% of the wines (Lasanta and Gómez 2012), and the efficiency of CMC in red wines was overall lower (Moutounet et al. 2010). All reports included a significant color loss that accompanied the occurrence of turbidity. Later experiments identified proteins as the main source of that haze (Claus et al. 2014; Sommer et al. 2016). The mechanism is described as a partially pH‐dependent reaction. At low pH, when the protein fraction holds a positive net charge and the carboxylic group on the CMC is protonated, the molecules can interact via a temporary dipole on the glucose backbone. At higher pH, the reaction is charge based, as the carboxy side chain of the CMC molecule holds a negative charge. This reaction mechanism also provides an explanation for the color loss that was observed in red wines. The protein fraction binds to anthocyanins, pigments, and tannins with a relatively low affinity. CMC, on the other hand, has a high affinity toward proteins and precipitates the polyphenols in combination with the proteins. There is no evidence that CMC reacts with polyphenolic material directly (Sommer et al. 2016).

While CMC has been successfully used in white and sparkling wines, it requires the same precautions in red wines. Proteins are usually removed from white wines using bentonite due to a higher level of visibility in the finished product (Sommer et al. 2015); however, since most red wines are aged and contain significantly more phenolic material, protein stabilization is usually not prioritized (Dordoni et al. 2015). This provides an explanation for why CMC can cause problems in red wines specifically. However, the haze formation was reported to be delayed by several days and even weeks after CMC addition (Sommer et al. 2016). This suggests that there are factors that can delay the interaction between these macromolecules in wine. The type of protein dictates the interaction itself (Claus et al. 2014), while other wine components influence the speed of aggregation. Factors that can affect the interactions of CMC with proteins include pH (Sommer et al. 2016) and the presence of other macromolecules such as polysaccharides (Sommer et al. 2019). Within the normal pH range of wine, low values around pH 3.0 and the higher range at pH 4.0 increase the affinity of proteins to CMC due to the different reaction mechanisms (Sommer et al. 2016). The role of polysaccharides and other macromolecules in this interaction is more complex. Most fining agents and additives seem to be more efficient in white wines compared to red wines (Sommer and Tondini 2021), which can be attributed to the skin maceration during red wine production and the resulting increase in the extraction of cell wall material. The overall net charge of that material determines the reactivity and which fraction of protein it shows an affinity toward (Ribéreau‐Gayon et al. 2021). Negatively charged polysaccharides show the strongest affinity toward proteins, while molecules with a positive charge will interact with polyphenols. A neutral molecule charge leads to an overall increase in particle size through agglomeration and an increase in haze formation independent of the wine pH (Sommer et al. 2019). It is worth noting that specific groups of polysaccharides do not follow these patterns, specifically the heterogeneous group of mannoproteins. They are described as protective colloids (Burken and Sommer 2025; Ribeiro et al. 2014; Rodrigues et al. 2012) and can slow or completely prevent the precipitation of macromolecules in wine. While this has been described for the formation of tartrate crystals (Lasanta and Gómez 2012) and the precipitation of protein (Ribeiro et al. 2014), in the presence of CMC, the effect of a protective colloid is weakened due to the strong affinity of CMC to proteins (Sommer et al. 2019). However, overall, the presence of polysaccharides could be the most influential factor for a delayed reaction between macromolecules in wine.

Understanding the mechanisms that are involved in the reaction between CMC and proteins has led to studies using the modified cellulose for achieving protein stability in wine (Sommer 2025; Sommer and Tondini 2021). While the use of CMC for selective protein removal was suggested earlier (Claus et al. 2014), a systematic study to evaluate the application in wine was still missing. With an average protein removal rate of 57% (Sommer 2025), the recent study showed the high potential of modified cellulose not only as an additive but also as a sustainable fining agent in wine. While regeneration studies of CMC for the use in food and wine do not exist due to the additive nature of the compound, its sustainability is apparent due to the biodegradability of used CMC (Kaur et al. 2023) in any winery waste stream.

### 4.4 CMC for Analysis

Due to the flexibility with molecular modifications and the resulting changes in hydrophobicity, solubility, and reactivity, CMC can be used as an analytical tool and reagent. Especially in the areas of enzyme activity assays, CMC shows properties that make it an interesting alternative to traditional methods (Johnsen and Krause 2014). Assays for the detection of endoglucanase activity (Xiao et al. 2005) and cellulase (Johnsen and Krause 2014; Sharrock 1988) are the most common examples. The gel‐forming properties of CMC are the most relevant characteristics that make it a flexible analytical tool.

Modified cellulose has also been used for selective adsorption of target compounds through solid‐phase extraction (Aladaghlo et al. 2024; Li et al. 2024). Especially its hydrophobic nature has made CMC a useful tool to target fats in food samples (Thabet et al. 2024; Thabet et al. 2023). That illustrates the potential for the analysis of consumer products such as food and wine. While the use of modified cellulose for the analysis of tannins in wine is explored thoroughly and described above, its use for the purification and analysis of proteins in wine is not described in the literature. While the use of CMC in reversible protein precipitation for characterization purposes is known (Lali et al. 2000; Zengin Kurt et al. 2017), its application in wine should be explored. Specific pathogenesis‐related proteins that lead to haze formation and decrease the physical stability of wine (Ferreira et al. 2001; Mierczynska‐Vasilev et al. 2017; Waters et al. 1996) could be purified and characterized using this isolation method. Other biomolecules such as chitosan that are used in wine (Bağder Elmacı et al. 2015) could improve the efficiency of protein purification and analysis (Sommer and Tondini 2021; Zengin Kurt et al. 2017).

## 5 Dicarboxymethyl cellulose

**FIGURE 4:**
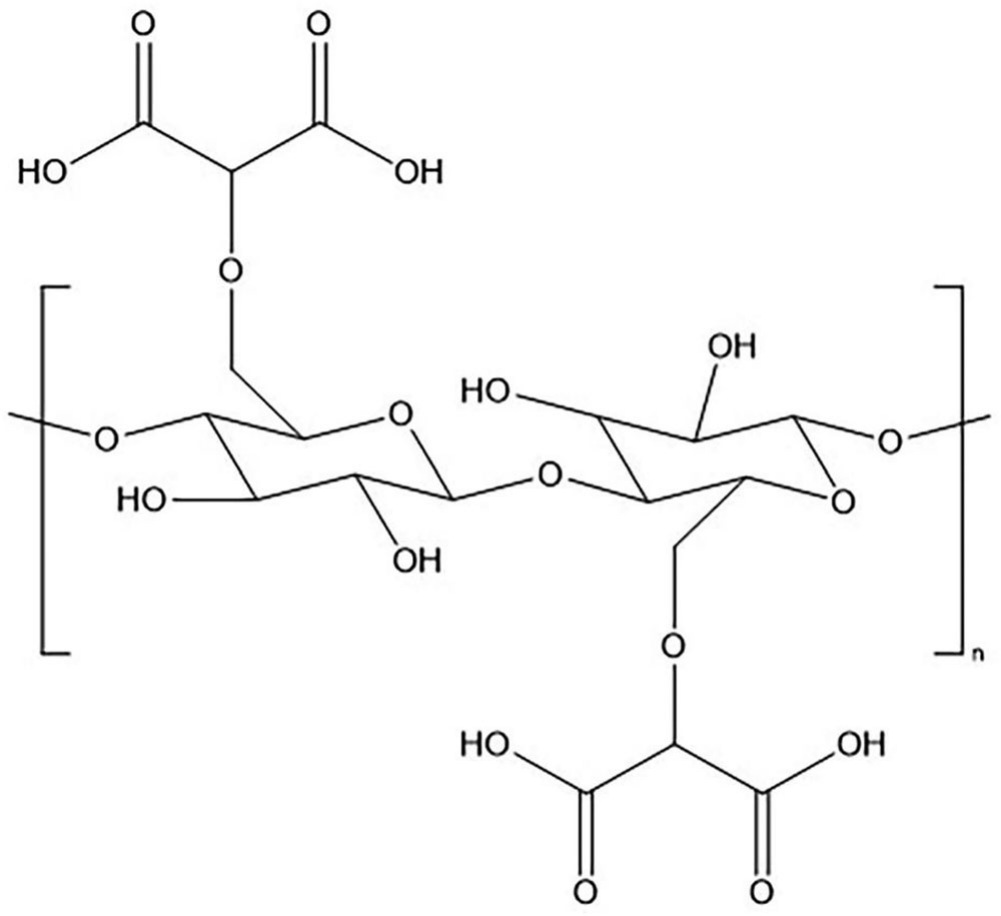
Molecular structure of a dicarboxymethyl cellulose subunit.

Purified cellulose can be modified to yield DCMC as another variation of soluble cellulose gums (Chagas et al. 2020). Similar to CMC, DCMC is used as a thickening or flocculating agent (Edwards‐Stuart 2021). The main difference from CMC can be found in its ionic properties and the high cation exchange capacity (Chagas et al. 2020; Gago et al. 2020). This makes DCMC especially useful in wastewater treatment, where the water is purified by flocculation and precipitation of contaminants (Coelhoso 2025; Sjahro et al. 2021). In some applications where metal ions need to be removed from the waste stream, DCMC can act as a chelating agent similar to CMC (El‐Saied et al. 1994), with some technological advantages over CMC due to the larger number of carboxylic acid groups. Figure 4 shows the molecular structure of a DCMC subunit. Most other applications of DCMC are related to its ability to thicken solutions; however, the use in food is less common than CMC. Most DCMC applications, except for their use in winemaking, are more industrial in nature, such as paints, detergents, and adhesives.

DCMC was used in recent years to specifically address protein stability in wine as an alternative to bentonite (Gago et al. 2021; Saracino et al. 2021). The dosage rate was comparable to that of bentonite in one study, and the addition achieved heat stability in the majority of wines (Saracino et al. 2021). Another study reported heat stability with addition rates above 0.25 g/L (Gago et al. 2021). While these additive concentrations are not excessively high and do not seem to influence the pH or aroma profile of the treated wines, the quantitative comparison to CMC reveals that CMC shows a higher protein removal efficiency at lower concentrations (Sommer 2025; Sommer and Tondini 2021). The reasons for that can be speculated to be related to the reactivity and three‐dimensional structure differences between CMC and DCMC. A recent study also demonstrated that DCMC can be regenerated and reused, which would make its use more sustainable than other fining agents (Gago et al. 2022).

## 6 Future Directions

While traditionally the focus for identifying additive and treatment options in wine has been on availability and functionality, the new trend toward sustainability is a strong argument for cellulose and its modifications. Not only is the regenerative nature of the raw material of this biopolymer appealing, but the modified cellulose can also be regenerated and reused, and after its lifespan, it is biodegradable. That shows all the elements of a technical production aid that meet the requirements of an environmentally conscious consumer. Due to the limited solubility of modified cellulose, it would still be considered an additive and would have to be listed as an ingredient on the label in some countries; however, cellulose is known to be a natural compound and would be more acceptable on an ingredient list than other treatment options.

With the few modification options presented in this review, the possible development opportunities become apparent. Not only can the cellulose itself be modified to change solubility, reactivity, and affinity toward specific groups of compounds, but it can also be used for filtration, for fining, and as an additive. Specific problems in wine production, such as off‐flavor removal and enhanced shelf life, can be addressed with a more targeted and sustainable approach, while overcoming some of the limitations of traditional fining agents and additives. Especially, the adsorption and selective removal of hydrophobic compounds such as tannins from wine is a promising application for modified cellulose to reduce astringency and improve physical stability of wine and other fermented products.

## Author Contributions

**Stephan Sommer**: conceptualization, investigation, visualization, writing – original draft, writing – review and editing.

## Conflicts of Interest

The author declares no conflicts of interest.
